# Two years' experience with Motorola Micromodules as an interface for a flame photometer

**DOI:** 10.1155/S1463924683000504

**Published:** 1983

**Authors:** R. A. Lutz

**Affiliations:** Med. chem. Labor Kantonsspital Winterthur CH 8401 Switzerland


Journal of Automatic Chemistry, Volume 5, Number 4 (October–December 1983), pages 204–206

### Two years' experience with Motorola Micromodules as an interface for a flame photometer

**R. A. Lutz**
*Med. chem. Labor, Kantonsspital, CH 8401 Winterthur, Switzerland*

### Introduction

Data transfer is one of the most critical operations in the laboratory. Online data acquisition offers several advantages, but hardware interfaces are often very expensive. Recent developments in microprocessor technology offer a flexibility which was not previously possible. In the author's laboratory results have to be entered either by hand or with a mark-sense card reader. Two Vitatron AKES analysers are linked to a minicomputer with a hardware interface. This accounts for about 40% of the routine work-load; another 10 to 15% are the electrolytes. Therefore the installation of online data acquisition to the flame photometers was desirable. The photometer has a BCD output, so an interface is needed to connect it directly to the main laboratory computer, which uses the standard RS-232C protocol on its multiplexer. With the microprocessor technology available today, an intelligent interface offers the best solution—a 'personal' computer could be used for this purpose since it gives the user the ability to make software changes easily and to adapt its program to meet changes in working conditions.

A different approach was chosen for the following reasons: firstly, the system had to be very reliable for routine working conditions. Disc-operating systems are fairly reliable, but it is still possible to damage a disc or to erase the information contained on it. This can be avoided by programmable read-only memories. Secondly, the system had to meet industrial standards and needed gold-plated connectors to withstand laboratory conditions—this is not always the case in personal computers because the price has to be kept low. Thirdly, it had to be precisely tailored to the specific application with a minimum of hardware development time—this is often impossible with personal computers because the possible hardware variations are limited. Fourthly, data loss had to be minimal: personal computers rarely have a RAM with battery back-up. Finally, it had to be fast and simple for a laboratory technician to use and once installed and working properly, no changes could be made. An interpreter language, which is most commonly used in personal computers, is therefore unnecessary.

These requirements were met in every respect by the use of subsystems based on an existing microprocessor (the M6800); the subsystems are known as 'Micromodules' [1]. With these micromodules it is possible to implement a specific system on a standard bus structure. It can be configured with central processing modules, memory modules and I/O ports. This concept offers the most economical approach for the application under discussion here, as only a small number of specific systems were going to be installed.

### Hardware

A Motorola M68MM01A monoboard microcomputer in a M68MMLC chassis was used. A Matsushita electrosensitive printer, series EUY-10E with a custom-made interface and a Model 753 keyboard (George Risk Industries, Inc., Kimball, Nebraska, USA), was directly linked to the peripheral interface adaptors (MC6820L) of the monoboard computer using shielded cables. The microprocessor is connected to the main laboratory computer (NOVA 3, Data General) through the asynchronous interface adaptor on the monoboard computer. An Input/Output Module Supplement from Motorola (MEX6820) was used to branch the IL flame photometer; Tri-State bus transceivers (DM 8833) from National Semiconductor were wire-wrapped to the outputs/inputs of this board. The flame photometer's printing signal was used to interrupt the microprocessor for data transfer; experience showed that a MC14490FL hex contact bounce eliminator was necessary to avoid causing any false interrupts. Six bounce eliminators were connected in series and a 680 pF capacitor was used to adjust the oscillator frequency. A relay was required to short the interrupt line to ground when the diluter was 'off'.

An M68MM09E CMOS-RAM module (Motorola) non-volatile random access memory was used to store the results; up to eight other microprocessors could be linked to it with MEX ACIA module supplements—so the microprocessor functions as a simple multiplexer. The basic system is illustrated in figure 1. In addition to the IL 343 flame photometer, three other instruments are shown on this figure to indicate the multiplexing capabilities of the system.

### Software

The software was written in the Motorola 6800 assembler language because no higher programming language was available at the start of the project. The program has editing capabilities; the major control loop is shown in figure 2. There are five possible entries and for each of these a letter is used as follows.

Typing 'L' (German: 'V' for Verzeichnis) after the ready sign '#', results in a list of all values and serum numbers stored in the memory being printed. Starting fresh, this is normally nothing but the first empty block. The microprocessor then prints 'NO SERUM NUMBER', together with the ready sign. If serum numbers have been entered previously, it prints them sequentially, together with the letter indicating whether it is a sample collected today, tomorrow or yesterday. If a result is stored with the serum number, the word 'PRESENT' is printed. With this program it is possible to check which of the serum numbers have been entered and whether a corresponding result is stored with it.

When 'C' (German: 'L' for loeschen) is typed after the ready sign, the whole memory can be cleared. This was necessary as some unwanted information was present in the memory during the initial testing of the system: a faulty PIA (peripheral interface adaptor) was the source of disturbance and was subsequently replaced. In order to be sure that the memory is only cleared when necessary, the message 'DO YOU REALLY WANT TO CLEAR THE WHOLE MEMORY?' is printed. The laboratory technician then has to answer with the letter 'Y' for yes. If another letter is typed then the program automatically exits

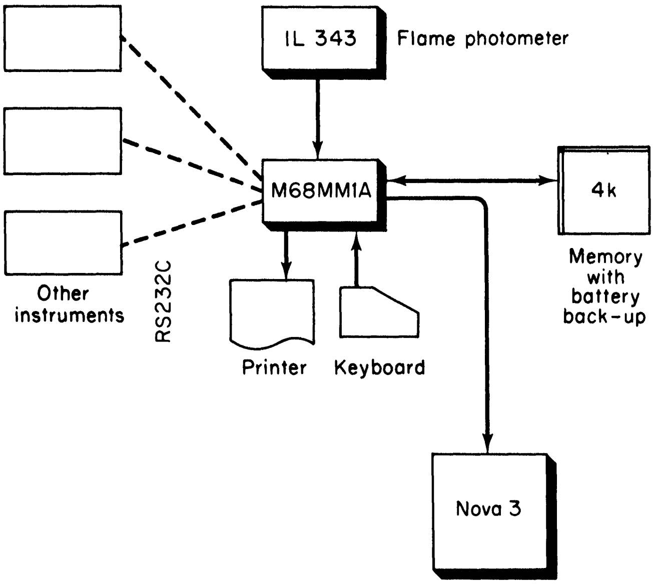

*Figure 1. Diagram of the microprocessor interface system. For further details see text.*

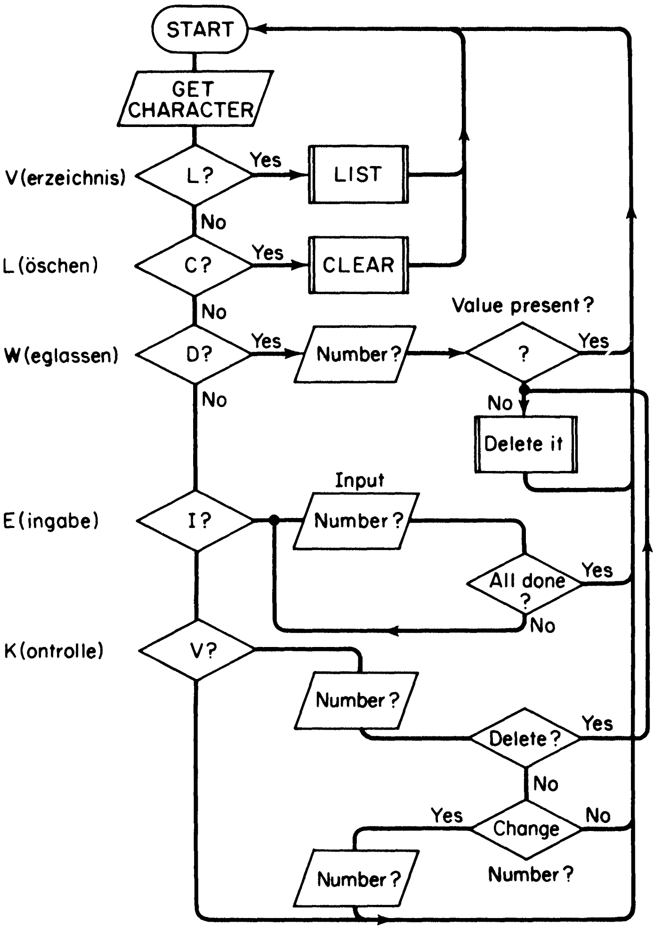

*Figure 2. Flow chart of the software. For further details see text.*

from this routine and nothing is changed. When the technician types 'D' after the ready sign (German: 'W' for weglassen), the 'DELETE' routine is called. With this routine it is possible to clear one serum number. As in the 'VERIFY' routine (see below) deleting is only possible when no result has been assigned to the serum number.

By typing 'I' for 'INPUT' (German: 'E' for Eingabe), the technician can enter the serum numbers; the computer system uses new serum numbers every day so it is necessary to specify the sample date. The new day begins in the middle of the afternoon, when all the results have been printed by the minicomputer. After each number, the operator can either press a carriage-return to proceed to the next number, or 'U' for urgent or 'E' for emergency samples. This information is stored in two bits, together with the serum number, and transmitted to the minicomputer when the results are obtained. If a mistake is made, typing a carriage-return instead of a serum number causes the program to exit this routine.

By typing 'V' (German: 'K' for Kontrolle) after the ready sign, the 'VERIFY' routine is called. This routine allows the operator to change the serum number or to delete it. Deletion is only possible if no reading has been assigned to it; this is for reasons of safety, as all the following serum numbers are moved one block forwards, which could cause misassignment of all the following values. If a value is assigned to a wrong serum number the whole entry has to be deleted (see above); re-entered and reanalysed.

During all these editing processes analyses can be carried out and transferred by pressing the 'DATA' button on the IL flame photometer, as the print signal interrupts the microprocessor. The serum number is then printed-out, together with the result, so that a hard copy of the result is kept. If an analysis is complete and no more serum numbers are present in the corresponding location of the memory, the message 'NO SERUM NUMBER' is printed. In this case, the result is also not stored. The analysis has therefore to be repeated after the corresponding serum number is entered.

Every time an analysis is performed the microprocessor checks whether the minicomputer is running and ready. If an acknowledge signal is not received from the minicomputer within 1 s of the results being transmitted, the microprocessor stores the data in its own memory with a battery back-up. In this case the microprocessor prints the message 'TRANSMISSION?' If an acknowledge sign is received, all the sera which have data assigned to them are transmitted. After successful transmission all the data results are cleared from the microprocessor's memory.

### Discussion

The requirements stated in the introduction were almost completely fulfilled. The system has been extremely reliable—no system breakdown has occurred during the last two years and no data have been lost since the faulty PIA was replaced. This is partly due to the fact that mechanical parts are kept to a minimum. Also, the program is stored in read-only memories—if a floppy disc or a similar storage device had been used then the reliability would most probably be reduced. In a laboratory environment where droplets and vapours of acids and organic solvents are present there are likely to be difficulties: corrosion of mechanical moving parts or dissolution of plastic materials have been observed by the author. Even though strong chemicals are now less common in clinical chemistry the risk should be kept to a minimum. One drop of a strong chemical on a floppy disc can completely ruin it. Reduced reliability can also be caused by the use of low-cost parts. The keyboard used in this example was cheap and did not have gold-plated connectors. No problems occurred with the system but a more reliable type is strongly recommended. It should be waterproof so that no corrosion can take place on the printed circuits if liquid is spilled on it.

The micromodules allowed a tailored system with minimal hardware development time to be built. One of the few hardware changes required was due to unexpected pick-up of electrical noise caused by the instrument and the instruments next to it. An attempt was made to circumvent this problem by checking the print signal of the flame photometer several times. Next, the whole system was shielded. As this did not completely cure the pick-up, the contact bounce eliminator (MC14490) was wire-wrapped between the print signal and the interrupt line of the microprocessor. The main switch of the dilutor also caused interference; since analyses cannot be carried out if it is turned off the microprocessor should not receive an interrupt signal through the data-line from the instrument. As an interrupt occurred whenever the dilutor was turned off, a relay had to be installed to the switch of the dilutor which forced the data-line to ground when no power was applied. More than half of the development time was used to circumvent the above difficulties.

The system has proved to be simple for the user: only one demonstration is needed to introduce laboratory technicians to the system. It was, however, observed that the laboratory technicians do not take full advantage of the capabilities of the system. Some routines were more often used than others—the INPUT routine was most frequently used, next were LIST, CLEAR and DELETE; VERIFY was least used.

The system is fast because there is no interpreter to slow down the functions of the interface. The use of an assembler programming language seems cumbersome; nevertheless it was highly educational—the programmer is forced to know the hardware and software in detail. Eight-bit microprocessors are not so complicated that the details cannot be overlooked by a part-time programmer. The system was developed within two months of the hardware arriving—programs were written during the free hours of the everyday routine work.

The assembler program listings are available from the author. The input routine could easily be adapted to a bar-code reader, which would allow a more positive sample identification. It was thought better to wait for an improved form of identification because the bar-code is still quite long and laboratory technicians cannot read it. With the advances in word-recognition systems, a more sophisticated approach might soon be possible.

### Acknowledgement

A first version of this paper was presented during a poster session at the 1982 Barcelona Congress on Automation in the Clinical Laboratory.

### Reference

1. Brugger, C., *The Micromodule Concept* (Motorola Semiconductor Product Division, Geneva, Switzerland, 1978).

### THE PITTSBURGH CONFERENCE

*5–10 March 1984 at Atlantic City, New Jersey, USA*

The call for Papers for the 35th Pittsburgh Conference was issued during June. Abstracts were due by 15 August—although those received after this date might still be included in the technical programme. The following symposia have been organized:

Spotlight on chromatography
Analytical techniques using supercritical fluids
Advanced light sources
Microprobe techniques as applied to organic materials
New techniques in electroanalytical chemistry
New opportunities in mass spectrometry
Sample introduction for plasma and flames: how can we do it better?
Integrating software into laboratory systems
Polymer characterization
Industrial hygiene monitoring
New horizons in nuclear magnetic resonance
The really sensitive techniques
ASTM E-42—Industrial applications of surface analysis
Pittsburgh analytical chemistry award
Pittsburgh spectroscopy award
Dal Nogare award symposium
The Williams-Wright industrial spectrocopist award

The symposium 'Spotlight on chromatography' will feature three invited symposia and an evening discussion group sponsored by the Pittsburgh Conference and organized by the International Meeting on Capillary Chromatography.

General papers are not restricted to symposia topics: they can be contributed in all areas of the disciplines of analytical chemistry and applied spectroscopy.

*The Continuing Education Programme*

1984's programme will contain, on 9 and 10 March, a short course on 'Hardware and software solutions to laboratory data management problems' (directed by Frank W. Plankey, Jr.), Mini short courses also on 9 and 10 March; and workshops on applications of personal computers on 8 March.

*Contacts*

Those wishing to read a paper should write to Mrs Linda Briggs (Department J-212) and enquiries about reserving space should be directed to George Vassilaros, *Pittsburgh Conference, 437 Donald Road, Pittsburgh, Pennsylvania 15235, USA.*

